# A Free-Space Optical Communication System Based on Bipolar Complementary Pulse Width Modulation

**DOI:** 10.3390/s23187988

**Published:** 2023-09-20

**Authors:** Jinji Zheng, Xicai Li, Qinqin Wu, Yuanqin Wang

**Affiliations:** 1 School of Electronic Science and Engineering, Nanjing University, Nanjing 210023, China; zhengjinji@smail.nju.edu.cn; 2 Electronic & Information Engineering, Nanjing University of Information Science & Technology, Nanjing 210044, China; wuqinqin@nuist.edu.cn

**Keywords:** free-space optical communication, modulation and demodulation, bipolar complementary pulse width modulation, multi-bandpass spectral subtraction

## Abstract

In this work, we propose a bipolar complementary pulse width modulation strategy based on the differential signaling system, and the modulation–demodulation methods are introduced in detail. The proposed modulation–demodulation strategy can effectively identify each symbol’s start and end time so that the transmitter and receiver can maintain correct bit synchronization. The system with differential signaling has the advantages of not requiring channel state information and reducing background radiation. To further reduce the noise in the system, a multi-bandpass spectrum noise reduction method is proposed according to the spectrum characteristics of the received modulation signals. The proposed modulation method has an error bit rate of 10^−5^ at a signal-to-noise ratio of 7 dB. The fabricated optical communication system can stably transfer voice and text over a distance of 5.6 km.

## 1. Introduction

The free-space optical (FSO) communication system has the characteristics of high transmission bandwidth, excellent confidentiality, and no electromagnetic interference [1,2,3]. Generally, according to the number of transmission channels, optical communication systems can be divided into single-channel and multi-channel. The most straightforward implementation of the FSO system scheme adopts single-channel intensity modulation and direct detection (IM/DD). To the best of the authors’ knowledge, the IM/DD system with non-return-to-zero (NRZ) modulation has a high dependence on the signal’s amplitude. However, the signal amplitude fluctuates as the distance changes, and fixed threshold detection is challenging to ensure long-distance communication and a higher decoding rate [4,5]. The adjusting or adaptive threshold value can effectively overcome the shortage of fixed threshold detection in NRZ demodulation [6,7,8]. X. M. Zhu et al. employed the maximum-likelihood sequence detection method for symbol detection to realize decoding. However, the scheme requires prior information about the transmission channel model, and the transmission channel model is susceptible to background light interference. Furthermore, the calculation algorithm of the automatic threshold has high complexity [6]. This research is extended by [7]. The authors proposed an improved maximum-likelihood sequence detection algorithm based on the single-step Markov chain model to reduce the complexity of the decoding algorithm at the receiving end. However, this algorithm is still highly dependent on priori parameters and requires channel state information. S. L. Ding et al. investigated an adaptive threshold decision scheme without the knowledge of channel state information [8]. However, in adaptive threshold detection, it is first necessary to collect and analyze the statistical characteristics of the received signal, such as noise level, signal strength, etc. Then, based on these statistical characteristics, the appropriate value of the adaptive threshold is calculated. This involves processing a large amount of data and complex mathematical operations, such as estimating signal parameters, regression analysis, statistical inference, etc. In addition, adaptive threshold detection may require multiple iterations to adjust the threshold to achieve optimal performance continuously. This means more time and computational resources are needed to complete this process.

Compared with the single-channel threshold detection method, the differential signal transmission system with two channels has a small amount of calculation, low threshold fluctuation, and a strong ability to reduce the impact of background radiation. It does not need channel state information and can greatly improve the system’s signal-to-noise ratio (SNR). Mohammad-Ali Khalighi et al. first proposed a differential signaling scheme to reduce the adverse impact of background radiation [9]. Based on this system, Mojtaba Mansour Abadi et al. verified that the optimal detection threshold level is 0, and a zero-detection threshold level could be employed to eliminate the effect of pointing error [10,11]. Li Xiaoyan et al. further studied the performance of the differential system. They considered the effects of partially correlated and fully correlated atmospheric turbulence attenuation in the differential transmission system, and they indicated that this system can avoid suffering from the “error floor” that plagues the FSO system with a fixed threshold [12]. Manav R. Bhatnagar applied quantized feedback to improve the system performance under uncorrelated atmospheric turbulence fading [13]. However, in the case of NRZ and RZ, if long strings of ones or zeros are transmitted, they would lose clock synchronization. This problem may be avoided with Manchester coding, in which it is possible to recover the clock of the digital signaling [14]. However, Manchester decoding requires edge detection of the signal, therefore requiring complex circuit design and implementation.

In this paper, we design a bipolar complementary pulse width modulation and demodulation strategy (BC-PWM) for the dual-channel free-space optical communication system and propose a multi-bandpass spectral subtraction denoising algorithm. Firstly, the structure of a dual channel free-space optical communication system is introduced, and the principle of bipolar complementary PWM is explained. Furthermore, to reduce signal noise, a multi-bandpass spectral subtraction denoising algorithm based on the frequency spectrum characteristics of the received signal is analyzed. Then, the BER performance of the proposed BC-PWM and the denoising performance of MBSS is compared with other methods, and we observe a significant improvement. Finally, we further perform the experiment in a physical system.

## 2. Principle of the Bipolar Complementary Pulse Width Modulation

**Figure 1:**
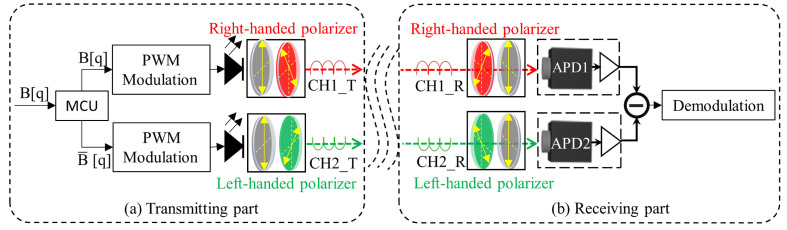
Proposed system block diagram. (**a**) Transmitting part; (**b**) receiving part.

The proposed system block diagram can be seen in Figure 1. Let us denote B[q] as the transmitted binary coded sequence (B[q]∈{0,1}). At the transmitting part, B[q] and its inverted version $\overset{-}{B}[q]$ are generated in the microcontroller unit (MCU) to modulate two-channel light sources to generate pulse width modulation PWM signals CH1_T and CH2_T, respectively. In order to carry out differential modulation for the above complementary two-channel signals, we use two circular polarization structures at the transmitter to realize the spatial separation. Because the polarization state of optical signals is almost unaffected by atmospheric disturbances, the transmission of the polarization state of optical signals can effectively improve the system’s ability to resist atmospheric disturbances. The use of linear polarization requires the alignment of the polarization coordinates of the transmitter and the receiver, which makes it difficult to guarantee the system performance of the FSO systems installed on moving objects [15]. At the receiving end, the CH1_R and CH2_R are converted into two electrical signals, $S_{1}$ and $S_{2}$, respectively, by the avalanche photodiode (APD) sensor, and the differential signal $S_{\mathrm{Diff}}$ of $S_{1}$ and $S_{2}$ is used to demodulate.

### 2.1. Modulation and Demodulation

**Figure 2:**
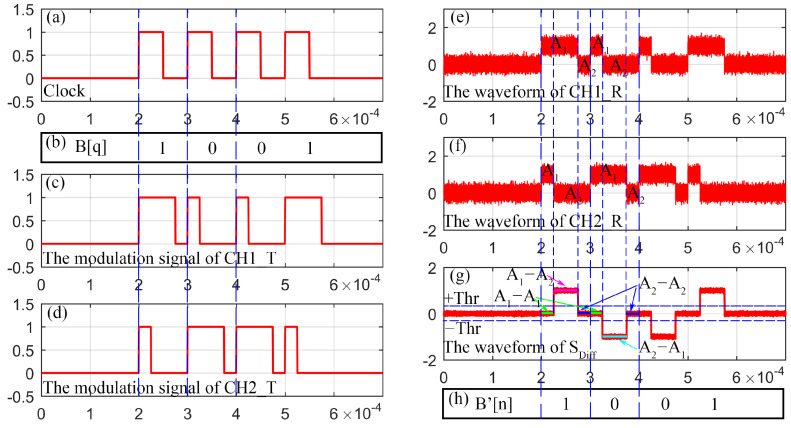
Schematic diagram of the modulation and demodulation signal. (**a**) The clock signal; (**b**) the sequence of the signal to be transmitted; (**c**) signal of CH1_T; (**d**) the complementary signal CH2_T; (**e**) the received signal in CH1_R; (**f**) the received signal in CH2_R; (**g**) the differential signal; (**h**) the sequence of the demodulation signal.

The schematic diagram of the modulation and demodulation signal is depicted in Figure 2.

The reference clock is shown in Figure 2a, assuming n is the index of the clock period. B[q] (q∈{0,1}) is defined as the symbol of the signal to be transmitted, as shown in Figure 2b. $D_{m}\left[q\right]$ is the duty cycle of the PWM signals in the $m_{\mathrm{th}}$ channel (m={1,2}), as shown in Figure 2c,d. When channel *m* is 1, the binary sequence B[q] is transmitted, and channel 2 transmits its converted version $\overset{-}{B}[q]$. $D_{m}\left[q\right]$ can be described by Equation (1):

(1) $$D_{m}\left[q\right]=\left(k_{m}^{q}+\frac{1}{4}\right)T_{B}\begin{array}{cc} , & q\in \left\{0,1\right\} \end{array},$$

where $k_{m}^{q}=q/2$.

$S_{m}(t)$ is the received signals output by the APDs in CH1_R or CH2_R, as shown in Figure 2e,f. Suppose $A_{1}$ and $A_{2}$ are the amplitudes of the received signals when the PWM signal is at a high level and low level, respectively. The $S_{m}(t)$ can be described in Equation (2):

(2) $$S_{m}(t)=\left\{\begin{array}{c} A_{1},\begin{array}{c} t\in \left[nT_{B},\left(n+k_{m}^{q}+\frac{1}{4}\right)T_{B}\right] \end{array}\\ A_{2},\begin{array}{c} t\in \left[\left(n+k_{m}^{q}+\frac{1}{4}\right)T_{B},(n+1)T_{B}\right] \end{array} \end{array}\right.\begin{array}{cc} , & n=0,1,2\ldots \end{array},$$

According to Equation (2), the differential signal $S_{\mathrm{Diff}}(t)=S_{1}(t)-S_{2}(t)$ can be described as Equation (3):

(3) $$S_{\mathrm{Diff}}(t)=\left\{\begin{array}{c} 0,\begin{array}{c} t\in \left[nT_{B},\left(n+\frac{1}{4}\right)T_{B}\right] \end{array}\\ A,\begin{array}{c} t\in \left[\left(n+\frac{1}{4}\right)T_{B},\left(n+\frac{3}{4}\right)T_{B}\right] \end{array}\\ 0,\begin{array}{c} t\in \left[\left(n+\frac{3}{4}\right)T_{B},(n+1)T_{B}\right] \end{array} \end{array}\right.,\;\begin{array}{c} n=0,1,2\ldots \end{array},$$

where $A=A_{1}-A_{2}$ or $A_{2}-A_{1}$. $A_{1}-A_{2}>0$ and $A_{2}-A_{1}<0$, so $S_{\mathrm{Diff}}(t)$ can be equivalent to a bipolar RZ signal. Set the threshold value as ±Thr, and the demodulated signal $B^{\prime }\left[q\right]$ can be obtained through the threshold detection method, as shown in Figure 2g,h. The mathematical description of this process is shown in Equation (4):

(4) $$B^{\prime }\left[q\right]=\left\{\begin{array}{c} 1,\begin{array}{c} \mathrm{if}\;A>+\mathrm{Thr},t\in \left[(n+\frac{1}{4})T_{B},\left(n+\frac{3}{4}\right)T_{B}\right] \end{array}\\ 0,\begin{array}{c} \mathrm{if}\;A>-\mathrm{Thr}, \end{array}t\in \left[(n+\frac{1}{4})T_{B},\left(n+\frac{3}{4}\right)T_{B}\right] \end{array}\right.,$$

The reason why the duty circle of the PWM signal in one clock period is set to $\left(1/4\right)T_{B}$ or $\left(3/4\right)T_{B}$ is that the positive or negative level duration of the differential signal can always be equal to $\left(1/2\right)T_{B}$ and in the middle of one symbol.

### 2.2. Multi-Bandpass Spectral Subtraction Denoising Algorithm

**Figure 3:**
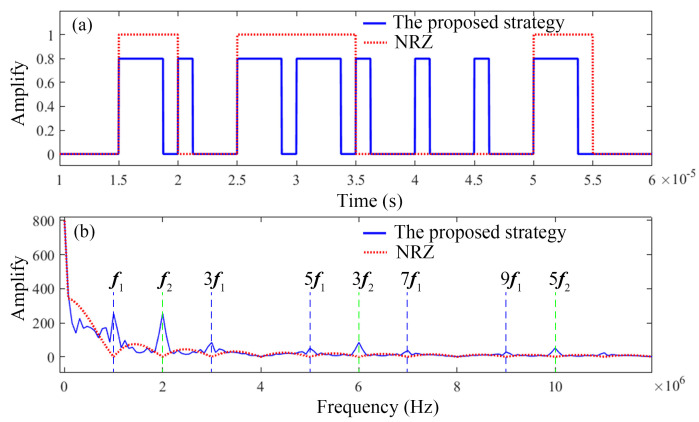
Diagram of the signals and frequency spectrums of the proposed strategy and NRZ modulation. (**a**) The ideal received signals for the proposed strategy and NRZ modulation; (**b**) the frequency spectrums of the received signals based on the proposed strategy and NRZ.

For the actual FSO communication system, the noise signal is unavoidable. To enhance the SNR, the denoise algorithm is necessary. The diagram of the signals and frequency spectrums of the proposed strategy and NRZ is shown in Figure 3.

It can be seen in Figure 3a that in the received signal based on NRZ modulation or other modulation methods, the duration of high and low levels is equivocal. For the proposed modulation strategy, the signal contains symbol 1 and symbol 0 with duty cycles of $\left(1/4\right)T_{B}$ or $\left(3/4\right)T_{B}$, respectively. Thus, the received signal can be approximately regarded as the combination of two kinds of square pulse signals. The frequency spectrums of the received signals based on the NRZ and the proposed strategy are shown in Figure 3b. The frequency spectrum of the received signal contains fundamental frequencies $f_{1}\;(f_{1}=1/\left(3T_{B}/4\right))$ and $f_{2}(f_{2}=1/\left(T_{B}/4\right))$ and their harmonic components. However, for the NRZ modulation, the characteristics of the frequency spectrum of the received signal are not outstanding. Therefore, with the proposed strategy, the multi-bandpass filtering [16] and multi-wind spectral subtraction [17] can be used to denoise the received signals. The specific filtering process is described as follows.

A signal can be regarded as the sum of the ideal signal and noise. Assume the received signal is y(n), which can be described by Equation (5):

(5) $$y(n)=x_{1}(n)+x_{2}(n)+N(n),$$

where $x_{1}(n)$ and $x_{2}(n)$ are the ideal square waves whose duty cycles are $\tau_{1}=\left(3/4\right)T_{B}$ and $\tau_{2}=\left(1/4\right)T_{B}$, respectively; N(n) is the noise signal.

The Fourier transform of y(*n*) can be expressed as Equation (6):

(6) $$Y(n)=\sum_{n=0}^{N-1}x_{1}(n)e^{-j2\pi k_{1}n/N}+\sum_{n=0}^{N-1}x_{2}(n)e^{-j2\pi k_{1}n/N}+\sum_{n=0}^{N-1}N(n)e^{-j2\pi k_{2}n/N},k_{1}=1,3,5\ldots ,\;k_{2}=1,2,3\ldots ,$$

Multi-bandpass filter design based on sectional combination complex modulated Gaussian function is used for filtering. The sectional combination complex modulated Gaussian function is given by [16]

(7) $$H(\Omega )=\sum_{k=1}^{K}\left(\frac{1}{C_{k}}\sum_{m=-M_{k}}^{M_{K}}e^{-[\sigma_{k}(\Omega -\Omega_{k,m}){]}^{2}/2}\right),$$

where H(Ω) is composed of $F_{1}(\Omega )$ with K segments, each segment has a $2M_{k}+1$ center frequency $\Omega_{k,m}$, and the interval is ${\Delta \Omega }_{k}$. The center frequency of each passband is $\Omega_{0,k}$, and the bandwidth is $2M_{k}{\Delta \Omega }_{k}$.

The passbands of the multi-bandpass filter are around $f_{1}$, $f_{2}$, ${3f}_{1}$, and ${3f}_{2}$. Thus, the harmonic component k=1,3 of $Y_{n}$ can be retained, and $Y_{n}$ can be described in Equation (8):

(8) $$Y(n)=\sum_{n=0}^{N-1}x_{1}(n)e^{-\frac{j2\pi \mathrm{kn}}{N}}+\sum_{n=0}^{N-1}x_{2}(n)e^{-\frac{j2\pi \mathrm{kn}}{N}}+\sum_{n=0}^{N-1}N(n)e^{-\frac{j2\pi \mathrm{kn}}{N}},\;k=1,\;3,$$

Noisy signal  y(n) is recorded as $Y_{i}(m)$ after windowing and framing. Signal $Y_{i}(m)$ is Fourier-transformed to obtain the phase spectrum of signal $\left|\theta_{i}(n)\right|$. Multi-window spectral estimation [17] is performed on signal $Y_{i}(m)$ to obtain multi-window spectral power spectral density P(n,i).

Smooth power spectral density $P_{y}(n,i)$ is obtained by smoothing between adjacent frames:

(9) $$P_{y}(n,i)=\frac{1}{2M+1}\sum_{j=-M}^{M}P(n,i+j),$$

Since the noise spectrum cannot be obtained directly, the estimated value $P_{n}(n)$ is calculated in the non-signal segment:

(10) $$P_{n}(n)=\frac{1}{\mathrm{NIS}}\sum_{j=1}^{\mathrm{NIS}}P_{y}(n,i),$$

The calculation of the gain factor through spectral subtraction relation can be described as:

(11) $$g\left(n,i\right)=\left\{\begin{array}{c} \begin{array}{cc} \left(P_{y}(n,i)-\alpha P_{n}(n))/P_{y}(n,i\right) & P_{y}(n,i)-\alpha P_{n}(n)\geq 0 \end{array}\\ \begin{array}{cc} \beta P_{n}(n)/P_{y}(n,i) & \;\;\;P_{y}(n,i)-\alpha P_{n}(n)<0 \end{array} \end{array}\right.,$$

where α is the over-minus factor and β is the gain compensation factor.

The amplitude spectrum after spectrum subtraction can be obtained through the gain factor and the average amplitude of the signal after multi-bandpass filtering:

(12) $$\left|{\hat{Y}}_{i}(n)\right|=g(n,i)\times \left|{\overset{-}{Y}}_{i}(n)\right|,$$

Finally, IFFT is performed through the amplitude spectrum and phase spectrum after spectrum subtraction, which can restore the frequency domain to the time domain and obtain the noise-reduced signal:

(13) $$\left|{\hat{y}}_{i}\left(m\right)\right|=\mathrm{IDFT}\left[\left|{\hat{Y}}_{i}(n)\right|\exp[j\theta_{i}(n)]\right],$$

Thus, for the proposed modulation–demodulation strategy, the multi-bandpass filter and spectral subtraction can denoise the received signal, and we name this method multi-bandpass spectral subtraction (MBSS).

## 3. Experiments and Analysis

We validated our method using three aspects. Firstly, a simulation analysis was conducted to compare the BER performance of the proposed BC-PWM with PPM and NRZ, as well as the denoising performance of the proposed multi-bandpass spectral subtraction and basic spectral subtraction. Secondly, a prototype was built to verify the feasibility of the proposed method. Finally, a full-duplex communication system was designed and a remote experiment was conducted.

### 3.1. BER and Denoising Algorithm Simulation Analysis

The efficiency of optical transmission is a decisive factor affecting the transmission distance of communication systems. The main optical components in optical communication systems include circular polarizers, transmission lenses, and transmitting and receiving lenses. For the designed optical communication system, the loss of optical devices is constant when the hardware parameters are fixed. Combining transmission path loss [18] and optical device loss, the final received optical power is represented by Equation (14):

(14) $$P_{r}=P_{t}L_{\mathrm{tpl}}L_{pl}L_{\mathrm{tl}}L_{rl},$$

where $P_{r}$ is the received optical power, $P_{t}$ is the transmitted power, $L_{p}$ is the circular polarizer with a loss of 47%, $L_{\mathrm{tl}}$ is the transmission lens with a loss of 3%, $L_{rl}$ is the receiving lens with a loss of 3%, and $L_{\mathrm{ls}}$ is the path loss, which is represented by Equation (15):

(15) $$L_{\mathrm{tpl}}={\left(\frac{\lambda }{4\pi d}\right)}^{2},$$

where λ is the wavelength and d is the path length. In close-range communication, the wavelength loss is relatively small and can generally be ignored. After the formation of the optical communication system, the transmission efficiency is mainly affected by the transmission loss of the transmission path. The following focuses on analyzing the impact of turbulence. Theoretical analysis based on measured values during the experimental process shows that the overall optical transmission efficiency of the entire system is approximately 47%. Due to the influence of alignment accuracy in actual systems and atmospheric turbulence in actual experimental processes, it is slightly lower than the theoretical value.

**Figure 4:**
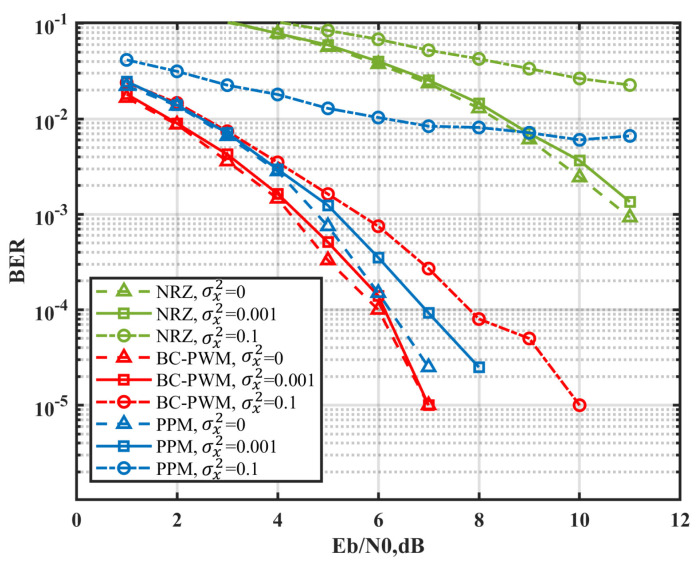
Bit error-rate (BER) against signal-to-noise ratio (SNR) for a range of $\sigma_{x}^{2}$.

The scintillation caused by atmospheric turbulence will lead to the optical loss. Atmospheric turbulence is generally equivalent to a Gaussian distribution model based on statistical averaging theory. The performance of BC-PWM is compared with NRZ and PPM in the Gaussian channel and log-normal atmospheric turbulence channel [19,20,21,22], as shown in Figure 4. The probability distribution function of log-normal distribution is given by [23]:

(16) $$f\left(I\right)=\frac{1}{I\sqrt{2\pi \sigma_{I}^{2}}}\exp(-\frac{\left(\ln(I\right)-\mu_{I}{)}^{2}}{2\sigma_{I}^{2}}),$$

Figure 4 shows the bit error rate (BER) performance with different background noise and logarithmic amplitude variance. The BC-PWM modulation strategy can suppress noise and atmospheric turbulence better than the NRZ and PPM modulation. The advantage is more evident with the increased logarithmic amplitude variance. This shows that the proposed BC-PWM strategy is more suitable for long-distance and high-light background environments.

**Figure 5:**
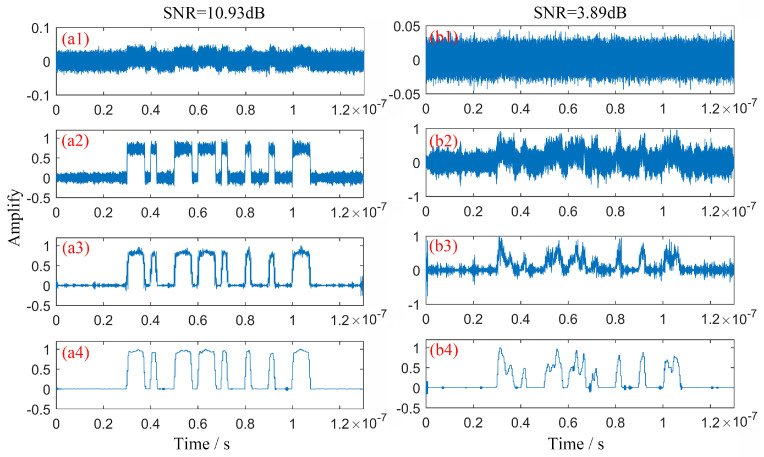
The denoising effect on signals. (**a1**) Simulation of received signal with SNR = 10.35 dB; (**a2**) signal denoised by BSS; (**a3**) signal denoised by MWSS; (**a4**) signal denoised by MBSS. (**b1**) Simulation of received signal with SNR = 3.49 dB; (**b2**) signal denoised by BSS; (**b3**) signal denoised by MWSS; (**b4**) signal denoised by MBSS.

Simulations were performed to verify the effect of the MBSS denoising algorithm on the received signal. The simulations set two received signals whose modulation modes are the same, and their SNR is 10.35 dB and 3.49 dB, respectively, as shown in Figure 5(a1,b1). From Figure 5(a2–a4), we can see that traditional basic spectral subtraction (BSS), multi-window spectral subtraction (MWSS), and multi-bandpass spectral subtraction (MBSS) significantly reduce the noise interference when the SNR is 10.35 dB. The denoising results are shown in Figure 5(b2–b4) for when the SNR decreases,. The proposed MBSS performs better than the BSS and MBSS in noise suppression, which can be attributed to the idea of multi-bandpass further suppressing the noise.

**Figure 6:**
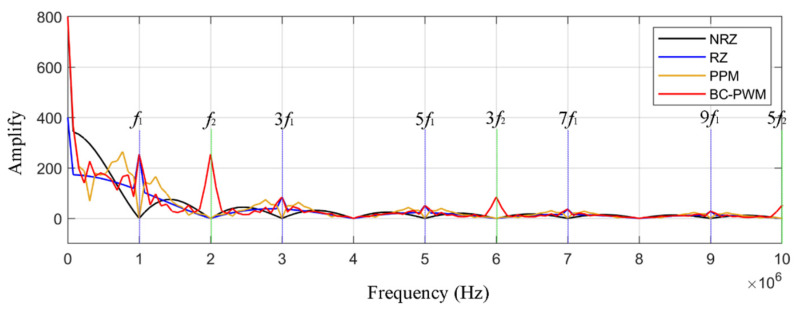
Spectral distribution of BC-PWM, NRZ, RZ, and PPM encoding methods.

At the same time, we analyze the spectrum of different encoding methods. The spectral distribution relationship of NRZ, RZ, PPM, and the method proposed in this paper is shown in Figure 6. The red curve in Figure 6 shows the spectral distribution curve of the encoding method in this paper. It can be seen that the signal amplitude at odd harmonics is significantly greater than that at other positions. The signal amplitude is significantly stronger than that of the RZ encoding method, which is more convenient for extracting more spectrum features and is conducive to multi-bandpass spectral subtraction (MBSS) denoising.

**Figure 7:**
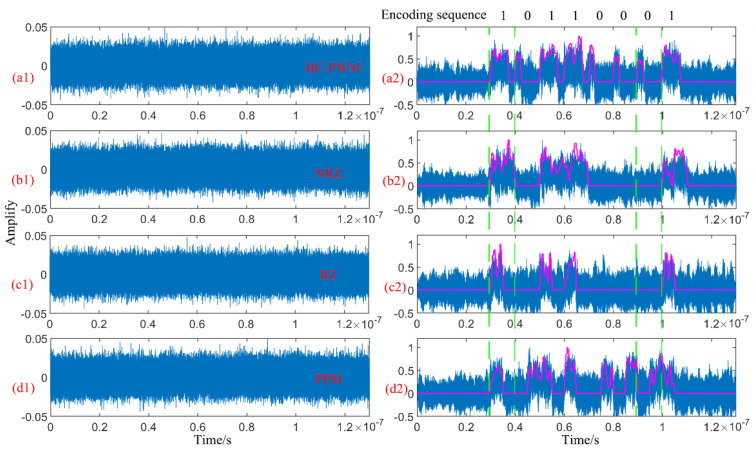
The denoising effect of using MBSS for BC-PWM, NRZ, RZ, and PPM encoding methods. (**a1**) The noisy signals of BC-PWM; (**a2**) the filtering effects of BSS and MBSS for BC-PWM; (**b1**) the noisy signals of NRZ; (**b2**) the filtering effects of BSS and MBSS for NRZ; (**c1**) the noisy signals of RZ; (**c2**) the filtering effects of BSS and MBSS for RZ; (**d1**) the noisy signals of PPM; (**d2**) the filtering effects of BSS and MBSS for PPM.

As shown in Figure 7, a comparative analysis is conducted on the denoising effects of four different encoding methods using MBSS. Figure 7(a1,b1,c1,d1) show the noisy signals of BC-PWM, NRZ, RZ, and PPM encoded signals, respectively. Figure 7(a2,b2,c2,d2) show the filtering effects of BSS and MBSS for four different encoded signals, with the blue curve representing the filtering effect of BSS and the magenta curve representing the filtering effect of MBSS. For the four types of encoded signals, MBSS has better denoising performance than BSS. However, NRZ-, RZ-, and PPM-encoded signals can eliminate most of the noise after MBSS denoising, but there is significant jitter at high levels. The amplitude of the jitter will bring a certain bit error rate. BC-PWM coding retains more frequency features, and the jitter amplitude at high levels is smaller, which is more conducive to reducing the bit error rate.

### 3.2. Principle Prototype and Experiment

**Figure 8:**
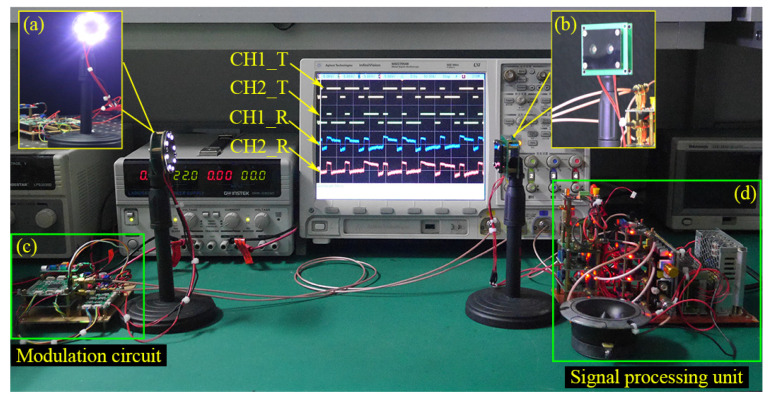
Physical map of the dual-channel FSO system. (**a**) The light sources; (**b**) the modulation circuit; (**c**) the APD signal receiving unit; (**d**) the signal processing unit.

According to the proposed BC-PWM strategy, a dual-channel FSO experiment setup was fabricated. The physical map of our dual-channel FSO system can be seen in Figure 8. The transmitting part contains a light source and a modulation circuit, as shown in Figure 8a,b. The dual-channel modulation signals are generated by the MCU (STM32f743, the main frequency is 200 MHz). The two groups of LEDs (2835, 0.2 W, 5500 Knm) can be driven to emit left- and right-hand polarized light, respectively (the left- and right-hand polarizers are assembled in front of two groups of LEDs, respectively). The receiving part contains two APDs (LSSAPD9-500-C1-NF-1-1) and a signal processing unit, as shown in Figure 8c,d. The right- and left-hand polarizers are assembled in front of two APDs so that the two-channel optical signals can be spatially separated. Thus, only polarized light of a specific polarization state can be received by each APD. Finally, the amplified signals of APDs are sent to the digital signal processing (DSP) unit for filtering and decoding.

**Figure 9:**
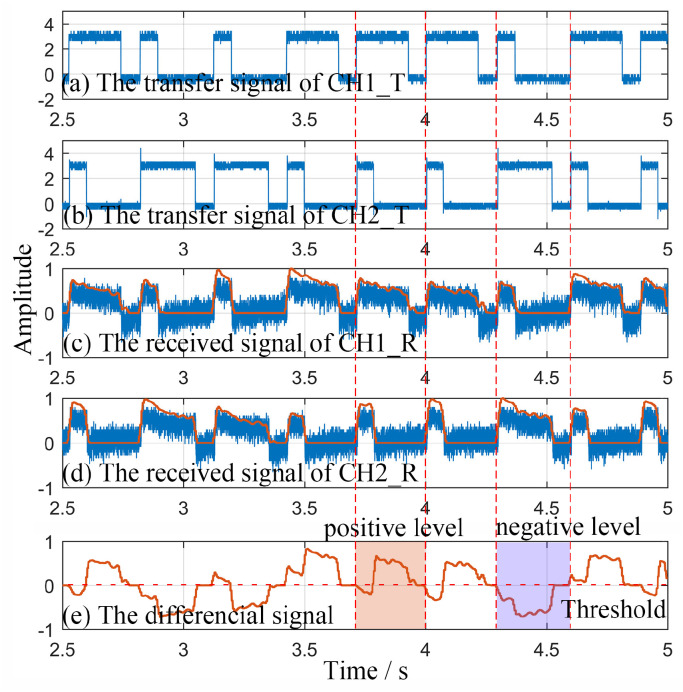
The signals in practical experiments. (**a**) Modulation signal in CH1_T; (**b**) modulation signal in CH2_T; (**c**) original (blue) and denoised (orange) received signals in CH1_R; (**d**) original (blue) and denoised (orange) received signals in CH2_R; (**e**) differential signal.

The generated BC-PWM signals can be seen in Figure 9a,b; the data rate is 40 kHz. The received initial signals output by APDs are shown as the blue waveforms in Figure 9c,d. As shown in Figure 9c,d, the left- and right-hand polarizers can effectively realize the spatial separation of modulation signal lights in two channels. The received signals denoised using the MBSS algorithm are shown as the orange waveforms in Figure 9c,d. The differential signal is shown in Figure 9e. According to the differential signal, we can realize the demodulation.

**Figure 10:**
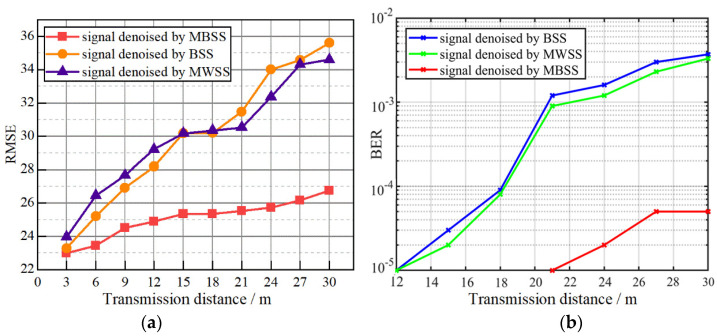
(**a**) The RMSE between the standard differential signals and the received differential signals denoised by MBSS, BSS, and MWSS; (**b**) the BER performance between the standard differential signals and the received differential signals.

The root mean square error (RMSE) is used to evaluate the property of our FSO system [24]. In experiments, we set ten different communication distances, and the received signals for different communication distances are recorded by the oscilloscope (Agilent MSO7054B 4 GSa/s). The received signals are denoised by the BSS, MWSS, and the proposed MBSS, respectively. Then, the final signal to be demodulated is obtained through a differential operation on the denoised dual channel signal. We take the differential signal of CH1_T and CH2_T as the reference signal and calculate the RMSE of the differential signals denoised by MBSS, BSS, and MWSS at different distances, as shown in Figure 10a. No matter which denoising method is used, the RMSEs always increase with the increase in communication distance. In addition, at various communication distances, the RMSEs of the proposed MBSS are always the smallest. This indicates that our MBSS can suppress noise better for the proposed BC-PWM strategy.

Further, the received signal was denoised using BSS, MWSS, and the proposed MBSS, and the bit error rate at different communication distances was analyzed. The BER performance is shown in Figure 10b. Compared to other denoising algorithms, the improvement in the maximum communication distance by using the MBSS algorithm is about nine meters when the bit error rate reaches 10^−5^.

### 3.3. Practical System Design and Remote Communication Experiments

**Figure 11:**
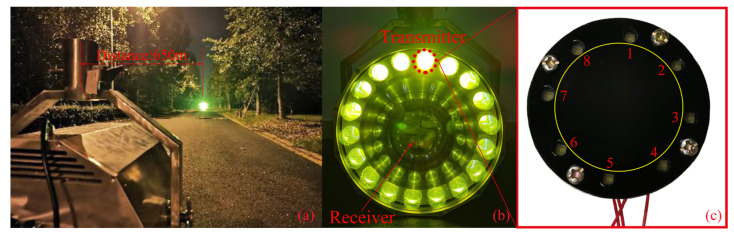
(**a**) Test scenario; (**b**) the full-duplex dual-channel FSO communication system; (**c**) the smallest transmitting unit.

The experimental link is located at both ends of a straight road on campus and is approximately 650 m long, as shown in Figure 11a. We conducted tests at different distances and found that voice and text transmission can be stably achieved at 5.6 km. The full-duplex communication system we designed is shown in Figure 11b, with the outer ring as the transmitter and the middle as the receiver. The transmitter consists of the 18 smallest transmitting units, as shown in Figure 11c, and contains a total of eight LEDs, with cardinal and even numbers grouped separately. Each set of LEDs is modulated by the proposed BC-PWM to obtain two channel signals, which are spatially separated by left-hand and right-hand polarization states.

In addition, due to the low complexity of the algorithm, the whole system has low requirements for the processor performance. After acquiring the signal at the receiving end, it successively goes through the steps of fast Fourier transform, MBSS filtering, signal difference, and decoding. No processes involve complex mathematical operations. For the coding process of the transmitter, we use STM32 interrupt implementation, so it does not consume CPU resources. With regard to the hardware cost, it mainly focuses on the light receiving sensor. In addition, it also includes the LEDs, light source driving circuit, polarization, STM32, and operational amplifier, which is characterized by low cost.

## 4. Conclusions

In this paper, we propose a BC-PWM modulation strategy for the FSO system, and the modulation and demodulation process are presented in detail. The performance of the BC-PWM is compared with NRZ and PPM in logarithmic turbulence conditions. With the logarithmic amplitude variance increased, the advantage of BC-PWM is more obvious. A zero-potential interval between adjacent pulses makes it easy for the receiver to identify each symbol’s start and end time so that the sender and receiver can maintain correct bit synchronization. Moreover, we propose a multi-bandpass spectral subtraction denoising algorithm according to the frequency spectrum characteristics of the received signals. The simulation experiments show that the MBSS has better denoising performances in comparison with the BSS and MWSS. Finally, the physical FSO system is fabricated to verify the performance of the MBSS denoising method in the BC-PWM strategy. With the light energy decreased, the RMSEs of MBSS are always the smallest compared with other denoising methods. This system has a strong noise suppression ability and can extract weak optical signals under high background noise. With optical power increases, the transmission distance would be longer, which can be helpful in applications for optical communication. Furthermore, a combination of space division multiplexing and wavelength division multiplexing can be used to enhance channel capacity.

## Data Availability

Not applicable.
